# Modulation of Bladder Wall Micromotions Alters Intravesical Pressure Activity in the Isolated Bladder

**DOI:** 10.3389/fphys.2018.01937

**Published:** 2019-01-10

**Authors:** Basu Chakrabarty, Dominika A. Bijos, Bahareh Vahabi, Francesco Clavica, Anthony J. Kanai, Anthony E. Pickering, Christopher H. Fry, Marcus J. Drake

**Affiliations:** ^1^School of Physiology, Pharmacology and Neuroscience, Faculty of Life Sciences, University of Bristol, Bristol, United Kingdom; ^2^Translational Health Sciences, Bristol Medical School, University of Bristol, Bristol, United Kingdom; ^3^Southmead Hospital, Bristol Urological Institute, Bristol, United Kingdom; ^4^Department of Applied Sciences, University of West England, Bristol, Bristol, United Kingdom; ^5^ARTORG Center for Biomedical Engineering Research, University of Bern, Bern, Switzerland; ^6^Department of Medicine, University of Pittsburgh, Pittsburgh, PA, United States

**Keywords:** bladder, bladder tone, gap junction, intravesical pressure, micromotions, phasic activity, spontaneous activity

## Abstract

Micromotions are phasic contractions of the bladder wall. During urine storage, such phasic activity has little effect on intravesical pressure, however, changed motile activity may underlie urodynamic observations such as detrusor overactivity. The potential for bladder motility to affect pressure reflects a summation of the overall movements, comprising the initiation, propagation, and dissipation components of micromotions. In this study, the influence of initiation of micromotions was investigated using calcium activated chloride channel blocker niflumic acid, and the effect of propagation using blockers of gap junctions. The overall bladder tone was modulated using isoprenaline. Isolated tissue strips and whole bladder preparations from juvenile rats were used. 18β-glycyrrhetinic acid was used to block gap junctions, reducing the amplitude and frequency of micromotions in *in vitro* and *ex vivo* preparations. Niflumic acid reduced the frequency of micromotions but had no effect on the amplitude of pressure fluctuations. Isoprenaline resulted in a reduction in pressure fluctuations and a decrease in pressure baseline. Using visual video data analysis, bladder movement was visible, irrespective of lack of pressure changes, which persisted during bladder relaxation. However, micromotions propagated over shorter distances and the overall bladder tone was reduced. All these results suggest that phasic activity of the bladder can be characterised by a combination of initiation and propagation of movement, and overall bladder tone. At any given moment, intravesical pressure recordings are an integration of these parameters. This synthesis gives insight into the limitations of clinical urodynamics, where intravesical pressure is the key indicator of detrusor activity.

## Introduction

Urinary bladders display spontaneous phasic activity during urine storage, both in terms of movements in the bladder wall (i.e., micromotions), and fluctuations in vesical pressure measured urodynamically (Vahabi and Drake, 2015). Spontaneous phasic contractions have long been known in tissue strips, but in whole bladder preparations it is possible to observe pressure fluctuations alongside the associated bladder wall movements (Drake et al., 2003a; Gillespie et al., 2015). The physiological relevance of this phasic activity is not established, but it may play a role in regulating the tone of the bladder, optimising the bladder to accommodate increasing volumes during storage, and influencing sensory afferent information (Iijima et al., 2009; McCarthy et al., 2009; Drake et al., 2017). Phasic pressure fluctuations have been observed urodynamically during the storage phase in several species of normal healthy animals, where they have been termed non-voiding contractions or non-micturition contractions (Andersson et al., 2011). There are changes in this activity upon bladder filling, especially when the volume in the bladder is high (Lagou et al., 2004; Biallosterski et al., 2011). There are also evident changes in rodent models of bladder disease (Drake et al., 2003b; Lagou et al., 2006a; Sekido et al., 2012; Wrobel et al., 2015). This activity has also been recorded in conscious, anaesthetised, or decerebrate animals (Pandita et al., 1998; Sadananda et al., 2013; Ito et al., 2018). Furthermore, it becomes enhanced in preparations when the brainstem is non-functional, suggesting centrally controlled inhibition of autonomous bladder wall contractions under normal physiological conditions (Sadananda et al., 2013).

In urodynamics, vesical pressure is a crucial parameter, which reflects detrusor contraction whilst allowing for increase in abdominal pressure. Increased detrusor pressure is indicative of a “bladder contraction,” which is a part of normal voiding. If it occurs in the storage phase, it would be termed DO (Abrams et al., 2002). The bladder is a micromotile structure; specifically, this means that observation of the bladder surface reveals localised contractions, and areas of quiescence, in a pattern that migrates over the surface with time. Micromotions may involve a varying amount of the bladder wall, and in some cases >50% of the total area (Drake et al., 2003b). Thus, bladder contraction may not always be a global contraction of the whole organ, rather a summation of the underlying phasic activity. It is proposed that the generation of small vesical pressure transients depends on the initiation and propagation of bladder wall micromotions. However, there needs to be sufficient overall tone in the detrusor muscle for the effect of the contraction to be transmitted to the contained volume, rather than being dissipated by elongation of quiescent parts of the bladder (Drake et al., 2017). If micromotions are initiated, yet confined to a small area, the effect on bladder pressure is likely to be negligible; hence the propagation of micromotions may be a key factor. Close inspection of micromotions indicates that the activity also includes localised elongations, and inactive areas with low intrinsic bladder tone, which could dissipate wall tension generated by micromotions, hence the importance of tone. Accordingly, at any given moment, detrusor pressure may reflect a summation of net force generation by micromotions, propagation, and the factors dissipating the force (Drake et al., 2017).

Bladder mucosa (consisting of the urothelium and suburothelium) plays an important role in mediating detrusor contractility. The suburothelium, which includes a population of interstitial cells, generates Ca^2+^ waves that modulate spontaneous contractions of neighbouring smooth muscle, and may be relevant in the initiation of micromotions (Kanai et al., 2007, 2011). Cl_Ca_ channel, Anoctamin-1, is co-expressed with interstitial cell markers in the rat urinary bladder, and Cl_Ca_ channel blocker, niflumic acid, reduced phasic activity (Bijos et al., 2014). The propagation of this electrical activity in the bladder is believed to be facilitated by gap junctions (Hanani and Brading, 2000; Hammad et al., 2014). Gap junctions are macromolecular structures that enable intracellular communication between cells. They allow free transfer of large and charged molecules (up to 1000 Da), including second messengers and ions (Spray and Bennett, 1985). They are formed by connexin (Cx) proteins in the cell membrane which are assembled as hexamers to form connexons that align with connexons in adjacent cells to form intracellular pathways (Geiger et al., 1995). In detrusor smooth muscle the main Cx subtype is Cx45 that forms gap junctions of relatively low electrical conductance. Thus, propagation of signals is relatively slow, when compared to, say, myocardium which is consistent with the slower propagation of spontaneous contractions. If micromotions are initiated, yet confined to a small area, the effect on bladder pressure is likely to be negligible; hence the propagation of micromotions may be a key factor. Close inspection of micromotions indicates that the activity also includes localised elongations, and inactive areas with low intrinsic bladder tone, which could dissipate wall tension generated by micromotions. Isoprenaline, a β-adrenoceptor agonist, results in the relaxation of bladder detrusor smooth muscle, and was used to assess whether motile and/or non-motile areas were affected. Accordingly, at any given moment, detrusor pressure may reflect a summation of net force generation by micromotions, propagation, and the factors dissipating the force (Drake et al., 2017).

In this study, we investigated the relationship between micromotions and intravesical pressure, by pharmacologically manipulating micro-contractions, and detrusor tone in isolated tissue strips and the whole rat bladder.

## Materials and Methods

### Tissue Preparation and Ethics Approvals

Urinary bladders were obtained from 21 ± 2 days old Wistar rats that were humanely euthanised by cranial concussion followed by cervical dislocation in accordance with the United Kingdom Animals Act (Scientific Procedures 1986). Bladders (with urethra attached for isolated whole organ preparations) were removed and placed in cold Krebs bicarbonate solution (118.4 mM NaCl, 11.7 mM glucose, 24.9 mM NaHCO_3_, 4.7 mM KCl, 1.9 mM CaCl_2_, 1.15 mM MgSO_4_, 1.15 mM KH_2_PO_4_ bubbled with a 95%O_2_:5%CO_2_ gas mixture to maintain a pH of 7.4).

### Isolated Tissue Strips

Bladders were opened by a ventral incision from the urethra to the dome. Each bladder was divided into two or three longitudinal strips. Strips of denuded detrusor were prepared by removing mucosa (urothelium and lamina propria) with blunt dissection under the dissecting microscope. Denuded (without mucosa) and intact strips (∼2 × 6 mm) were mounted in 0.2 mL Perspex microbaths (Oxford University, United Kingdom) and were superfused with carboxygenated Krebs (36 ± 1°C) at a flow rate of 2 mL.min^-1^ with a peristaltic pump. Strips were equilibrated under a resting tension of 1.0–1.5 g for 60 min. Tension was monitored with isometric force transducers (Pioden Controls Ltd., United Kingdom) connected to a Powerlab data acquisition system running LabChart software (ADInstruments, United Kingdom). To enhance the spontaneous contractions and increase the frequency of the denuded (muscle only) strip rhythmic activity, agents increasing muscarinic receptor stimulation were added. Carbachol (CCh, 10 μM) (Sigma-Aldrich, United Kingdom) directly stimulates muscarinic receptors and promotes muscle contraction. An initial priming response was elicited in all intact and denuded detrusor strips by exposure for 10 s to CCh dissolved in Krebs solution (Bijos et al., 2014). After a 10-min washout period, a 30-min control period of phasic contractile activity was recorded. A single dose of a gap junctional uncoupler [carbenoxolone (CBX, Sigma-Aldrich, United Kingdom) or 18β-glycyrrhetinic (18β-GA, Sigma-Aldrich, United Kingdom)] or the drug vehicle DMSO (Sigma-Aldrich, United Kingdom) was superfused for 30 min, with at least a 30-min washout period between the doses.

The effect of blocking gap junctions was investigated by measuring the amplitude (g tension per mg of tissue weight) and frequency (number of contractile events in 5 min) of phasic contractions. The amplitude and the frequency of phasic contractions were measured before (baseline) and during the last 5 min of any drug (experimental) or vehicle (control) exposure and compared using paired two-tailed Student’s *t*-test. Percentage inhibition (I%) of amplitude and frequency were calculated by subtracting the value at baseline from experimental and dividing it by values at baseline.

$$I\%=\frac{\left(\text{experimental-baseline}\right)}{baseline}\times 100$$

Comparisons between intact and denuded tissues at the same drug concentration were performed using an unpaired Student’s *t*-test. All data are presented as mean ± SEM. The null hypothesis was rejected at *p* < 0.05. Statistical analyses were performed in GraphPad Prism (GraphPad Software Inc., United States).

### Isolated Whole Organ

#### Experimental Protocol

**FIGURE 1:**
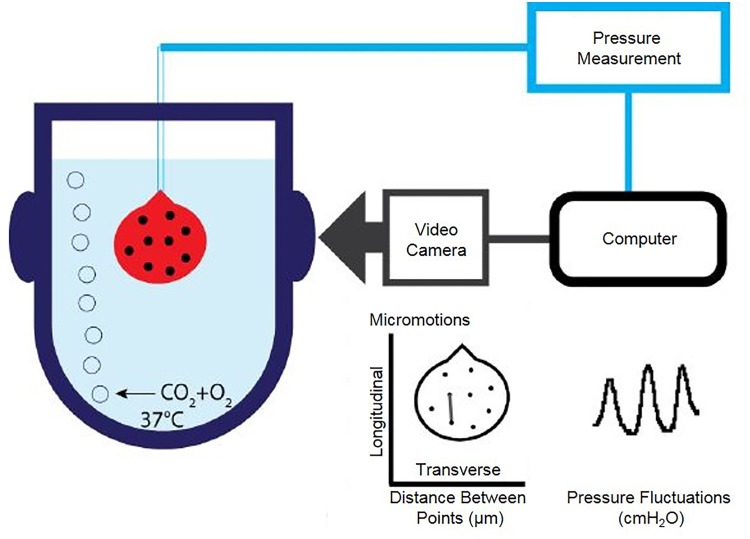
Schematic diagram of isolated whole organ apparatus recording pressure and bladder wall movements simultaneously. Catheterised bladder is placed upside down in carboxygenated buffer in a jacketed chamber held at 37°C. Pressure transducer attached to catheter is connected to an analogue to digital converter. Filming of the bladder is synchronised to pressure recordings.

A 26GA venflon (Becton Dickinson) attached to a 1 mL syringe filled with Krebs was inserted into the bladder via the urethra and secured with a thread above the vesico-ureteric junction. The bladders were filled slowly up to ∼350 μL and carbon particles were applied to the bladder surface to allow monitoring surface movements. The bladder was then placed in an organ bath (with an optical window for filming) containing carboxygenated Krebs solution, and maintained at 37°C. A camera (Prosilica EC650) connected to the custom written LabView application (National Instruments, United States) allowed simultaneous acquisition of pressure and video data at 10 frames per second (Figure 1). The bladders were allowed to equilibrate for a minimum of 15 min. Bladder pressure and movement were then inspected; if contractions were undetectable, 50–100 μL Krebs solution was added to the intravesical volume. The bladder’s position was adjusted to maximise movement visible to the camera. After at least 30 min of equilibration in isovolumetric conditions, pharmacological agents were added into the bath. Gap junction blockers 18β-GA and CBX, Cl_Ca_ channel blocker niflumic acid (Sigma-Aldrich, United Kingdom), β-adrenoceptor agonist isoprenaline (Sigma-Aldrich, United Kingdom) to reduce overall bladder tone, or the vehicle control were incubated with the tissue for 30 min.

Three parameters of bladder pressure traces were analysed: amplitude, frequency, and baseline pressure of spontaneous phasic contractions. Pressure fluctuations were detected at an amplitude threshold of 1 cmH_2_O above baseline. Five-minute periods before and after drug addition were compared with a two-tailed paired Student’s *t*-test. Raw values were plotted and percentage change of each parameter after drug addition was calculated. Distances between the carbon particles in the focused field of view on the bladder wall were analysed using an in-house custom LabView application programme and plotted in LabChart (ADInstruments, United Kingdom), and transferred to Adobe Illustrator, where final figures were assembled.

#### Video Data Analysis

To identify movement more easily, videos of bladders were watched at double speed. Initial visual inspection was used to determine presence or absence of movement. Quantification of the bladder wall movement over time was performed using custom made LabView software. The software analysed change in the distance between two points on the bladder wall over the time of the recording.

In LabView software the point search area was set (region of interest), then the contrast of the carbon particles was set to maximise the connexion between the points on the bladder wall and calibration was conducted to convert the distances between points, initially expressed in pixels into micrometres (μm). Software then followed a chosen distance over time for the selected duration of the recorded video, the distance was synchronised with the pressure recording. A file that resulted had pressure, and distance change over time. For each bladder, between 6 and 16 pairs of points were manually curated for validity and analysed. The pressure and distance traces were then visualised using LabChart (ADInstruments, United Kingdom) software and then combined in Adobe Illustrator. To see how movement timing relates to the pressure changes, the off-set of the distance from pressure change was calculated. The pressure fluctuation maximum value was taken as a pressure peak. The time in seconds between pressure peak and the distance of shortening peak was noted and plotted in LabChart (ADInstruments, United Kingdom).

## Results

### Spontaneous and Stimulated Phasic Activity

Rhythmic contractions induced with CCh in denuded and intact strips had a greater amplitude and a more consistent frequency compared to control spontaneous contractions (Student’s unpaired *t*-test, *p* < 0.05) (Supplementary Figure S1).

#### Isolated Denuded Tissue Strips

**FIGURE 2:**
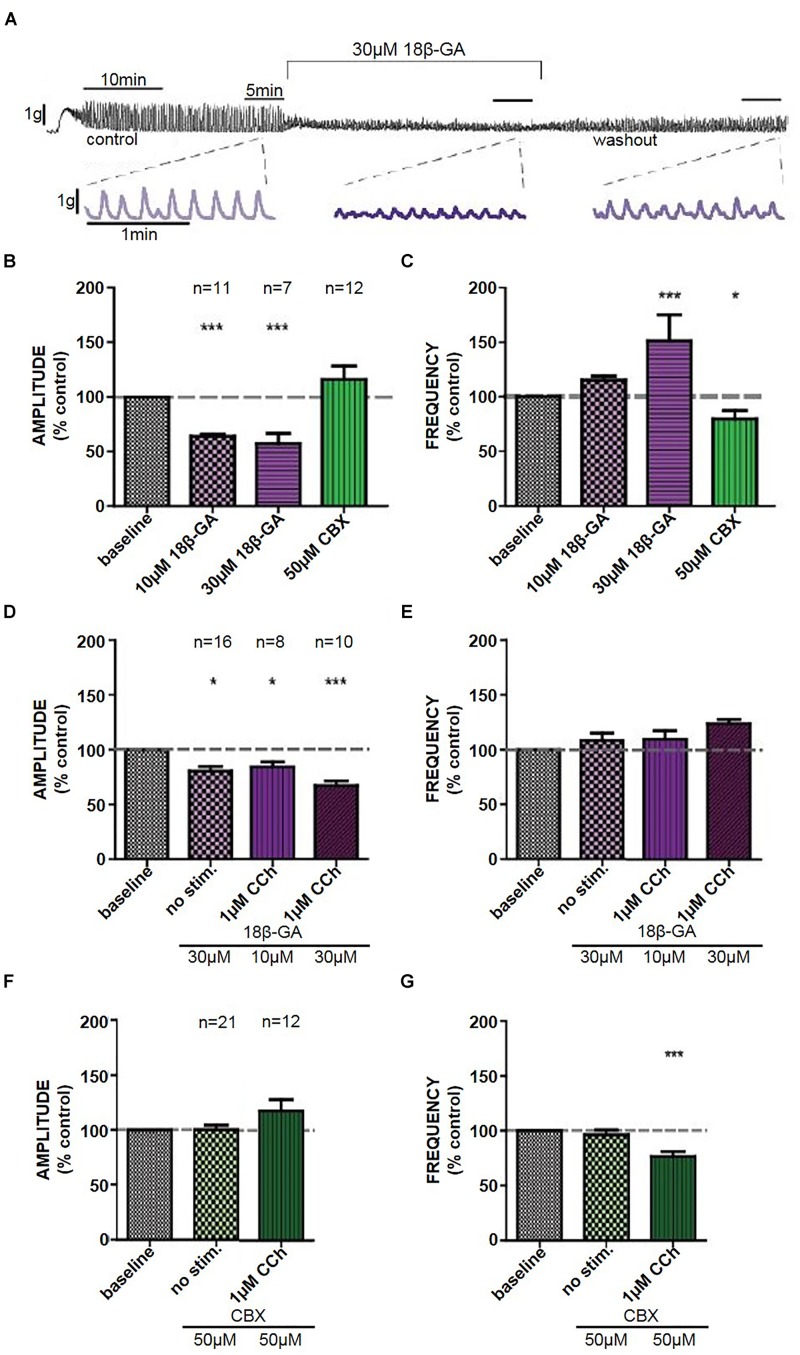
**(A)** Representative trace of the effect of 18β-GA (30 μM) on CCh-induced contractions in denuded strips, including an insert of the control, drug, and washout. The effects of 18β-GA (10 and 30 μM) and CBX (50 μM) in denuded strips on the **(B)** amplitude and **(C)** frequency of CCh-induced contractions. The effects of 18β-GA (10 and 30 μM) in intact strips on the **(D)** amplitude and **(E)** frequency of spontaneous and CCh-induced contractions. The effects of CBX (50 μM) in intact strips on the **(F)** amplitude and **(G)** frequency of spontaneous and CCh-induced contractions. ^∗^*P* < 0.05, ^∗∗∗^*P* < 0.001; Student’s paired *t*-test.

18β-GA (10 μM) decreased the amplitude of CCh-induced phasic contractions (Student’s paired *t*-test, *n* = 11, *p* < 0.001), whereas 18β-GA (30 μM) decreased the amplitude and also increased the frequency of CCh-induced phasic contractions (Student’s paired *t*-test, *n* = 7, *p* < 0.001) (Figures 2A–C). These effects were not restored back to control upon a 30-min washout period. CBX (50 μM) decreased the frequency of contractions (Student’s paired *t*-test, *n* = 12, *p* < 0.05) (Figure 2C). DMSO (0.1%) had no significant effect on CCh-induced contractions in isolated denuded tissue strips.

### Isolated Intact Tissue Strips

18β-GA (30 μM) decreased the amplitude of spontaneous (Student’s paired *t*-test, *n* = 16, *p* < 0.05) and CCh-induced (Student’s paired *t*-test, *n* = 10, *p* < 0.001) phasic contractions (Figure 2D). There was no significant effect on the frequency of phasic contractions in comparison to control (Figure 2E). CBX (50 μM) had no effect on spontaneous phasic contractions (Student’s paired *t*-test, *n* = 21, *p* > 0.05). CBX decreased frequency of CCh-induced phasic contractions (Student’s paired *t*-test, *n* = 12, *p* < 0.001) but had no effect on the amplitude of contractions (Figures 2F,G).

### Whole Organ Pressure Measurements

**FIGURE 3:**
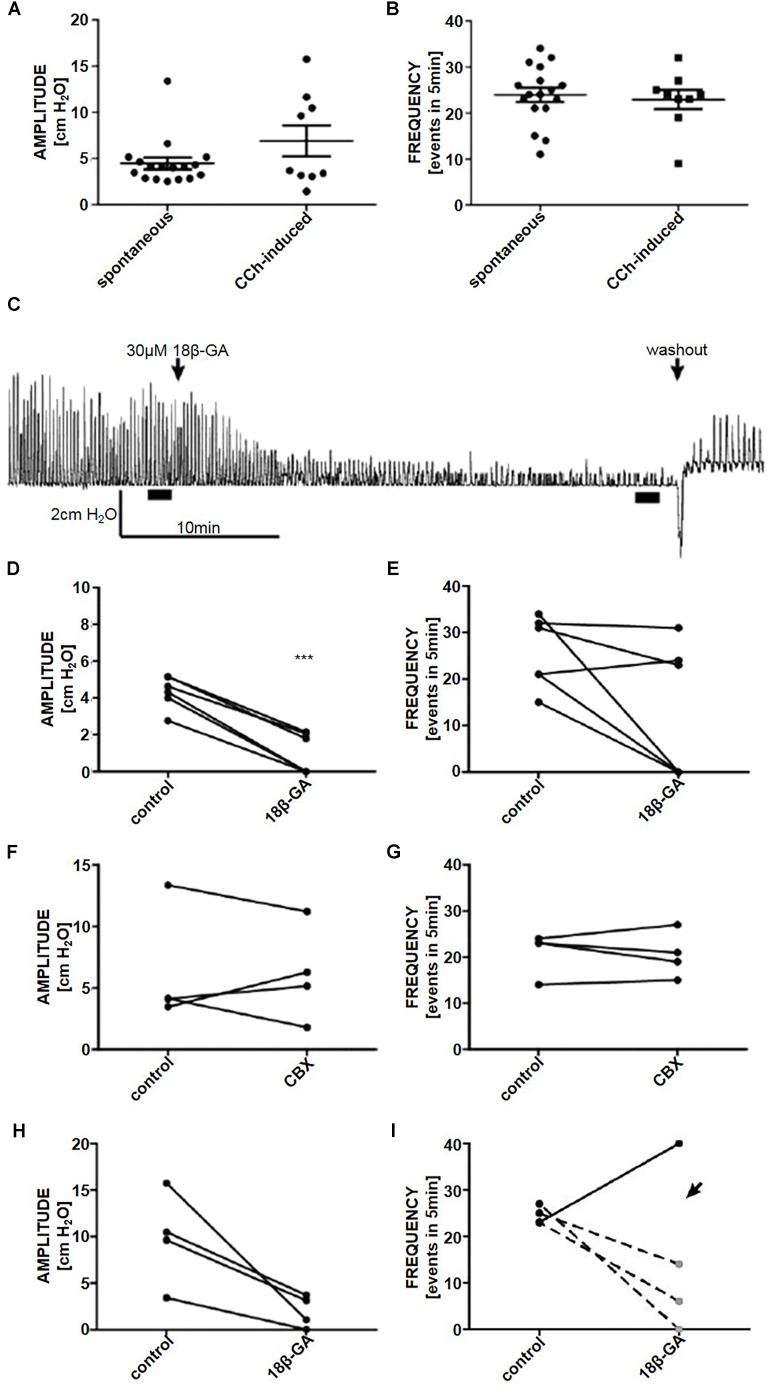
The **(A)** amplitude and **(B)** frequency of spontaneous (*n* = 17) and CCh-induced (*n* = 9) pressure fluctuations in an isolated whole rat bladder. **(C)** A representative trace of the effect of 18β-GA (30 μM) on spontaneous phasic activity of the whole bladder and the effects on **(D)** amplitude and **(E)** frequency of spontaneous phasic activity (*n* = 6). The effect of CBX (50 μM) on the **(F)** amplitude and **(G)** frequency of spontaneous phasic activity of the whole bladder (*n* = 4). The effect of 18β-GA (30 μM) on the **(H)** amplitude and **(I)** frequency of CCh-induced phasic activity of the whole bladder (*n* = 4). ^∗∗∗^*P* < 0.001; Student’s paired *t*-test.

A volume of 350 μl gave reproducible spontaneous phasic pressure fluctuations of 4.5 ± 0.6 cmH_2_O amplitude and 23.9 ± 1.5 events in 5 min (*n* = 17). These were not significantly different from CCh-induced pressure fluctuations [6.9 ± 1.7 cmH_2_O and 22.9 ± 2.1 events in 5 min (*n* = 9) (Figures 3A,B)].

#### Spontaneous Activity

18β-GA (30 μM) decreased the amplitude of spontaneous phasic pressure fluctuations by 79.8% (Student’s paired *t*-test, *n* = 6, *p* < 0.001) (Figures 3C,D). The frequency remained unchanged in three preparations where fluctuations were detectable (>1 cmH_2_O amplitude) (Figures 3C, 2E). CBX (50 μM) had no effect on the amplitude and frequency of spontaneous phasic pressure fluctuations (Student’s paired *t*-test, *n* = 4, *p* > 0.05) (Figures 3F,G).

#### Stimulated Phasic Activity

To stimulate whole organ phasic pressure fluctuations CCh (1 μM) was added. After the initial tonic contraction, phasic activity developed and was stable for the duration of the experiment (at least 2 h). 18β-GA significantly reduced the amplitude of CCh-induced phasic fluctuations (Student’s paired *t*-test, *n* = 4, *p* < 0.05) (Figures 3H,I).

The effects of 18β-GA and CBX on whole organ spontaneous and stimulated phasic pressure fluctuations are listed in Table 1.

**Table 1:** The effects of 18β-GA and CBX on rat whole bladder spontaneous and CCh-induced phasic pressure movements.

		Amplitude (% control)	Frequency (% control)
Spontaneous phasic pressure movements	18β-GA (30 μM) *n* = 6	20.21 ± 9.1^∗∗∗^	47.56 ± 21.9
	CBX (50 μM) *n* = 4	108.49 ± 29.5	98.39 ± 6.9
	DMSO (0.1%)	105.9 ± 0.0	100.0 ± 0.0
CCh-Induced pressure movements	18β-GA (30 μM) *n* = 4	18.64 ± 8.9^∗^	66.17 ± 40.5
	DMSO (0.1%)	102.67 ± 12.7	87.50 ± 8.3


*^∗^*P* < 0.05; Student’s paired *t*-test. ^∗∗∗^*P* < 0.001; Student’s paired *t*-test.*

### Bladder Micromotion Analysis

Each bladder was observed during recording of baseline activity and 30 min after addition of 18β-GA. In all experiments using 18β-GA, the pressure fluctuation amplitude was reduced. Spontaneous fluctuations were reduced below the detectable limit (<1 cmH_2_O) in three experiments, and in all of them visible movement of the bladder wall persisted despite the lack of detectable pressure changes (*n* = 4). Similar results were obtained in CCh-stimulated bladders; 18β-GA decreased the amplitude, yet movement was visible in 75% of bladders (*n* = 4).

#### Longitudinal Bladder Wall Movement

**FIGURE 4:**
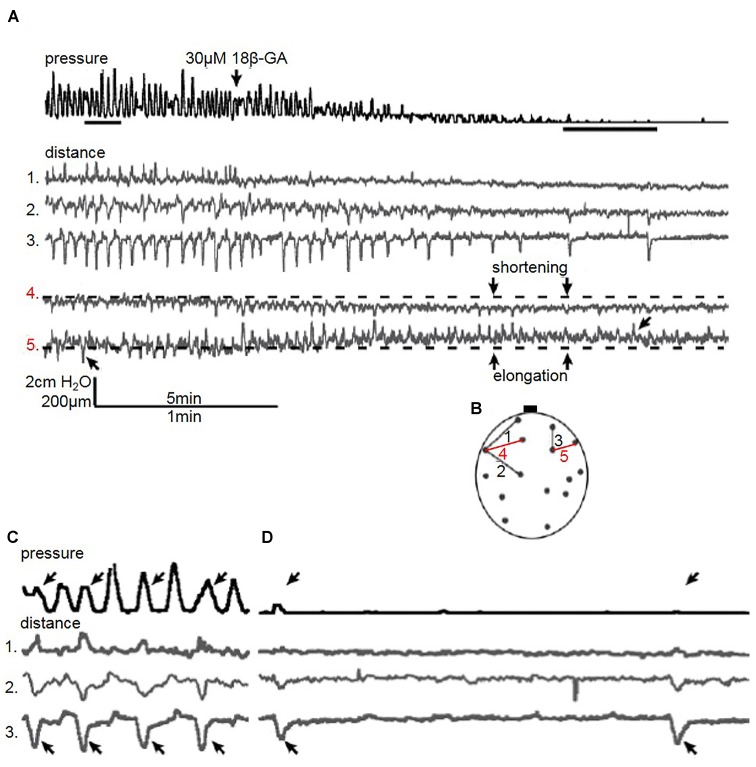
**(A)** A representative trace of the effect of 18β-GA (30 μM) on spontaneous pressure fluctuations in an isolated whole rat bladder, and the effect on longitudinal (distances 1, 2, and 3) and transverse (distances 4 and 5) components. Distance 4 shortens whilst distance 5 elongates, as demonstrated with respect to baseline – arrows in distance 5 demonstrate shortening and elongating events. **(B)** Schematic diagram depicting measured longitudinal and transverse distances. **(C)** Control spontaneous pressure fluctuations – arrows in pressure and distance 3 highlight pressure changes displayed by the shortening of distance 3. **(D)** The effect of 18β-GA (30 μM) on spontaneous pressure fluctuations – arrow in pressure and distance 3 demonstrate shortening movement of distance 3 without measurable pressure change.

Quantification of distance in the bladder wall demonstrated an altered pattern of movement when gap junctions were blocked with 18β-GA (30 μM). In the bladder in Figure 4, distances on the bladder wall with a substantial longitudinal component elongated and shortened, which coincided with pressure fluctuation changes (Figure 4A – distance 4 and 5). Not all pressure fluctuations can be explained by visible movement. Every other pressure fluctuation can be explained by localised bladder movement, suggesting local contractions in other bladder areas influenced pressure change (Figure 4C). Although 18β-GA abolished pressure fluctuations completely in three spontaneously contracting bladders, movement was present. Longitudinal distance number 3 was shortening despite the lack of visible pressure change (Figure 4D).

**FIGURE 5:**
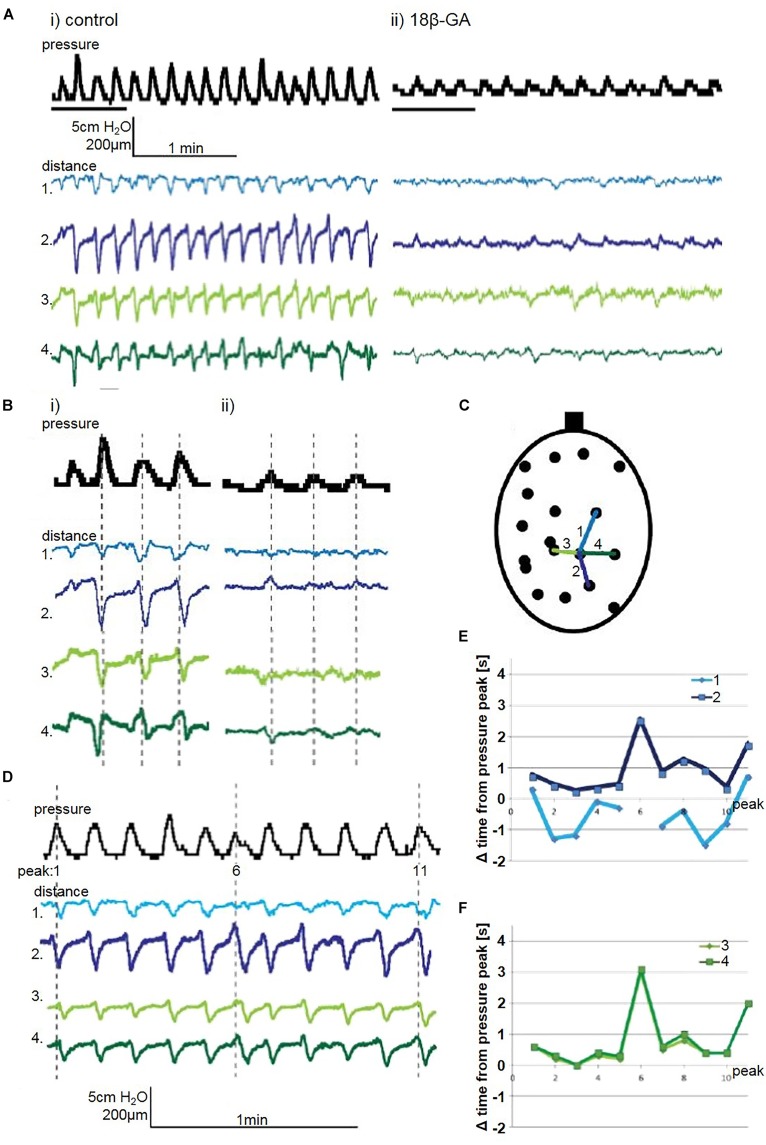
**(A)** A representative trace of spontaneous pressure fluctuations: **(i)** control and **(ii)** 18β-GA (30 μM), in an isolated whole rat bladder, and the effect on longitudinal (distances 1 and 2) and transverse (distance 3 and 4) components. **(B)** Enlargement of representative trace: **(i)** control and **(ii)** 18β-GA (30 μM), with grey dashed lines indicating the peak of pressure change, and relationship to distance change. **(C)** Schematic diagram depicting measured longitudinal and transverse distances. **(D)** A representative trace of spontaneous pressure fluctuations, and distances on the bladder wall with dominant longitudinal (distances 1 and 2) and transverse (distances 3 and 4). The offset of the distance shortening peak from the pressure fluctuation peak measured in seconds for **(E)** longitudinal and **(F)** transverse distances.

In the bladder in Figure 5, two consecutive longitudinal distances on the bladder wall are displayed – distance 1 and 2. Distance 1 is shortest just before peak of pressure. Microcontractions and microelongations of the bladder wall possessed rhythmicity which was not always synchronous with pressure change. Movement spreading along the longitudinal was observed where distance 1 and 2 showed a correlation of movement (Figure 5Bi).

#### Transverse Bladder Wall Movement

Movement observed during visual inspection was mainly in the longitudinal direction. However, as bladder pressure fluctuations became less pronounced after blocking gap junctions, the behaviour of bladder micromotions changed. Transverse (horizontal) distances are shown in Figure 4. Movement quantification revealed that overall shortening or elongation of the specified area could occur (Figure 5A – distance 4 and 5).

When movement spread in the longitudinal axes, the bladder in the transverse direction was affected. The rhythmical shortenings and elongations occurred in the transverse direction and reflected either an area where longitudinally spreading movement arrive (Figures 5E,F) or an area surrounding a focal point of non-propagating contractions (Figure 7E).

#### Spreading Movement

In spontaneously active whole bladders, as in Figure 5, the movement initiation centres were localised at the top of the bladder dome and bladder base (in superior and/or inferior sections of the bladder) and spreading vertically (longitudinally). These observations were quantified by measuring the time difference between the pressure peak and the shortenings of distance traces corresponding to the pressure change. These were measured by evaluating consecutive longitudinal and transverse distances in the spontaneously contracting bladder. In the measured area, the movement was observed mostly after the pressure change. Longitudinal distances showed a pattern of contractions, where one part was contracting before the other (Figure 5E), suggesting propagation of movement. Transverse distances were contracting at the same time (Figure 5F).

### Initiation of Micromotions

**FIGURE 6:**
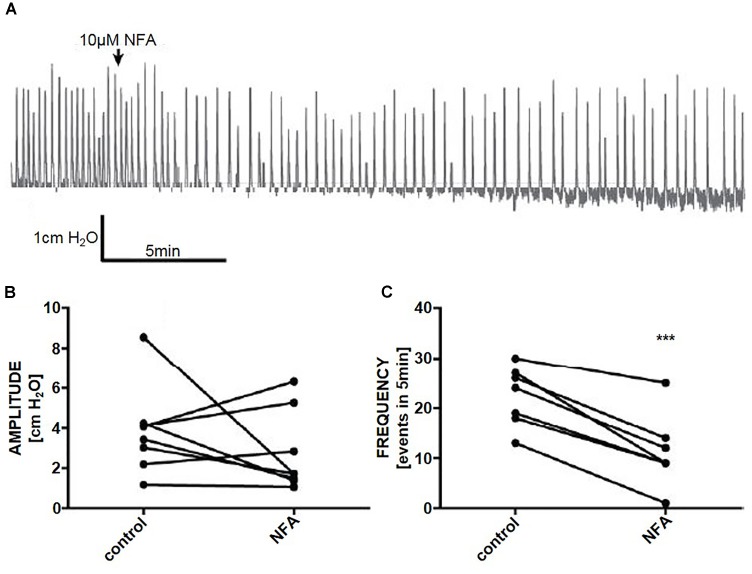
**(A)** A representative trace of the effect of niflumic acid (NFA. 10 μM) on spontaneous pressure fluctuations in an isolated whole rat bladder, and the effects on **(B)** amplitude and **(C)** frequency of spontaneous phasic activity (*n* = 6). ^∗∗∗^*P* < 0.001; Student’s paired *t*-test.

In whole organ preparations, niflumic acid (10 μM) reduced the frequency of phasic pressure fluctuations by 53% (Student’s paired *t*-test, *n* = 8, *p* < 0.001), however, there was no effect on the amplitude of phasic pressure fluctuations (*p* > 0.05) (Figures 6A–C).

### Whole Organ Relaxation

**FIGURE 7:**
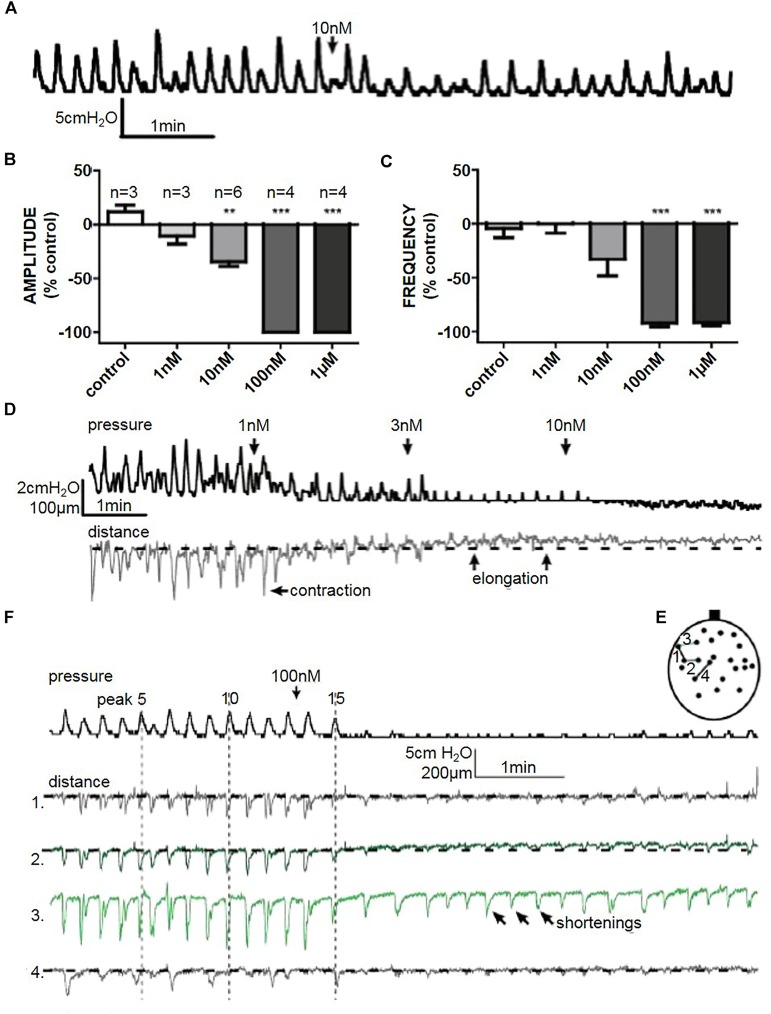
**(A)** A representative trace of the effect of isoprenaline (10 nM) on spontaneous whole bladder pressure fluctuations, and the effects on **(B)** amplitude and **(C)** frequency of spontaneous phasic activity. **(D)** The effect of isoprenaline on pressure and distance measured in filmed isovolumetric ex-vivo whole rat bladder. **(E)** A representative trace of the effect of isoprenaline (100 nM) on spontaneous pressure fluctuations and four distances on the bladder in filmed isovolumetric ex-vivo whole rat bladder. Shortenings correlated to pressure changes (distances 1, 2, and 4), and abolished in the absence of pressure change. Arrows demonstrate shortening movement without measurable pressure change in distance 3. **(F)** Schematic diagram depicting measured distances. ^∗∗^*P* < 0.01, ^∗∗∗^*P* < 0.001; Student’s paired *t*-test.

Isoprenaline reversibly reduced the amplitude of spontaneous pressure fluctuations in a dose dependent manner. 100 nM and 1 μM isoprenaline abolished spontaneous pressure fluctuations completely in a reversible manner within 30 s of addition of the drug to the bath (*n* = 4) (Figures 7B,C). 10 nM isoprenaline caused a reduction in spontaneous pressure fluctuation amplitude by 34.4 ± 5% (Student’s paired *t*-test, *n* = 6, *p* < 0.01), without significantly affecting frequency (Figures 7A–C). All doses reduced the baseline pressure of the bladder (Figure 7D).

At lower doses it was evident that, as pressure baseline decreased, parts of the bladder stopped contracting (shortening) and started to elongate. With 100 nM and 1 μM isoprenaline there was a quick (<30 s) cessation of pressure fluctuations (Figure 7E). Although the movement mostly decreased when isoprenaline stopped pressure fluctuations (Figure 7E – distance 1, 2, and 4), it did not completely block micromotions (Figure 7E – distance 3), suggesting that initiation is not blocked by isoprenaline. More likely, the overall tension of bladder wall decreased making the localised microcontractions smaller.

## Discussion

The findings shed light on the relationship between bladder micromotions and vesical pressure fluctuations. We observed congruent alterations in pressure fluctuations when we modulated initiation and propagation of micromotions with Cl_Ca_ channel and gap junction blockage, and tone with an adrenoceptor agonist.

### Phasic Activity in Bladder Strips and Whole Organ

Phasic activity of the bladder enhances with increasing volume (Lagou et al., 2006a; Sadananda et al., 2011). With *in vitro* preparations, phasic activity is observed by tensioning of tissue strips (Uvelius, 2001; Komari et al., 2013). Bladders with low volumes have less pronounced spontaneous phasic activity (Lagou et al., 2006b; Sadananda et al., 2011). In the current study, maximal volumes retrieved at the time of death were used as an indicative volume for undertaking whole organ pressure measurements as previously described (Drake et al., 2003a).

Pharmacological stimulation is used to enhance spontaneous contractions in isolated strips and whole organ preparations (Szell et al., 2003; Lagou et al., 2006b; Ng et al., 2006), and has also been used in different age groups and pathological conditions (Vahabi et al., 2011; Vahabi et al., 2013). In this study, the increase in amplitude of contractions is significant between spontaneous and stimulated *in vitro* preparations, as observed previously in similar age groups and concentrations used (Szell et al., 2003; Killi et al., 2014).

In isolated bladder strips, the force and amplitude of phasic contractions change during the first weeks of development (Szell et al., 2003). In this study, 3-week-old animals were used, with a bladder capacity of ∼400 μl (the mean bladder volume at time of death), displaying spontaneous pressure fluctuations of ∼5 cmH_2_O. Differences from previously reported studies using neonatal bladders may be due to varying ages (ranging from 3 to 21 days) (Ikeda et al., 2007). During postnatal development, key maturation changes of bladder signalling pathways occur (Szigeti et al., 2005). Bladder innervation and signalling matures, and involvement of higher autonomic control increases, whilst peripheral mechanisms become less evident (Ikeda et al., 2007; Kanai et al., 2014).

### The Gap Junction Blocker 18β-GA Reduces Bladder Phasic Activity

Gap junction blockers have previously been used on bladder tissue (Hashitani et al., 2001, 2004; Christ et al., 2003; Miyazato et al., 2006; Ikeda et al., 2007; Kim et al., 2011; Babaoglu et al., 2013). Similar effects have been found with 18α-GA and 18β-GA isomers (Miyazato et al., 2006; Ikeda et al., 2007; Kim et al., 2011). Reported effects of 18β-GA include reduced electrical coupling between bladder smooth muscle cells (Hashitani et al., 2001, 2004), as well as reduced contractions of bladder strips (Kim et al., 2011; Babaoglu et al., 2013). However, the effect of gap junction blockers in neonatal strips have not been previously characterised. Both 18α-GA (data not shown) and 18β-GA isomers reduced isolated strip phasic activity amplitude (Figures 2A–E). The effects of 18β-GA displayed strong congruence between *in vitro* and *ex vivo* findings. Pressure fluctuations in *ex vivo* whole organ preparations were decreased with 18β-GA in neonatal bladder (Figures 3A–E,H,I), similar to previous studies, albeit at a slightly higher dose (Ikeda et al., 2007). Higher concentrations to exert a similar effect may be needed due to the presence of fewer gap junctions in the bladder during the first few weeks after birth, suggesting that gap junction coupling may be important for the generation of high amplitude pressure fluctuations (Ikeda et al., 2007; Kanai et al., 2014).

### The Gap Junction Blocker CBX Has No Effect on Bladder Phasic Activity

*In vitro* strips and *ex vivo* whole organ experimentation showed no effect of 30-min. exposure to CBX (50 μM) on spontaneous phasic activity of the neonatal bladder (Figures 2F,G). However, CBX decreased the frequency of CCh-enhanced strip phasic activity (Figure 2G), suggesting that pacemaking/rhythmicity of phasic activity might be altered.

CBX (30 μM) blocked Ca^2+^ transients spreading in the guinea-pig bladder smooth muscle and interstitial cells (Hashitani et al., 2004). In mice, the same concentration of CBX reduced L-type Ca^2+^ channel opener (Bay K8644) induced contractions (Okinami et al., 2014). In the urothelium, CBX (100 μM) has a very small effect on urothelial ATP release (Sui et al., 2014). Circular smooth muscle cells from the guinea pig ileum lost responsiveness when gap junctions were uncoupled by CBX (100 μM) (Carbone et al., 2012). This may suggest that low concentration of CBX (50 μM) may not sufficiently exert an effect to be detected at the level of tissue contractions in spontaneous contracting preparations. In brain tissues, reported CBX effects on gap junction are varied, with main factors contributing to high variability in results including drug concentration and exposure time to the drug (Goldberg et al., 1996; Kohling et al., 2001; Yang and Michelson, 2001).

### The Cl_Ca_ Channel Blocker Niflumic Acid Reduces Phasic Pressure Fluctuation Frequency

Anoctamin-1 is expressed in interstitial cells in young rat bladders (Bijos et al., 2014), and niflumic acid modulates Anoctamin-1 and other Cl_Ca_ channels in the bladder (Li et al., 2008). In this study, niflumic acid reduced phasic pressure fluctuations in the whole bladder (Figures 6A–C), similar to observations in previous studies using tissue strips from the rat bladder (Bijos et al., 2014). The reduction in the frequency of phasic pressure fluctuations, but the lack of effect on the amplitude, suggests that modulating Cl_Ca_ channel activity affects the initiation of micromotions in whole bladder preparations.

### Relaxing Effects of β-Adrenoceptor Agonist

In human and rat bladder strips, isoprenaline acts as a β-adrenoceptor agonist (Rouget et al., 2014). *Ex vivo* whole organs were sensitive to isoprenaline; even very low concentrations (1–10 nM) had a fast-acting effect on phasic activity (Figures 7D,E). A decrease in bladder voiding parameters was seen previously in a working brain-stem preparation in the same animal age group (Sadananda et al., 2013). In the whole organ studies, the relaxation of the muscle is evident by the overall decrease in pressure baseline, and this may be indicative of the change in bladder tone.

### Movement Is an Important Component of Bladder Behaviour

Characterising phasic activity changes in denuded and intact bladder strips, and the whole organ, helps translate from simpler to more complex systems, enabling insight into the role of different components in the whole organ function (Chun et al., 1989; Kullmann et al., 2014). However, the presence of intra- and inter-bundle interstitial cells, may have a crucial role in muscle activity (Hashitani et al., 2004; Gray et al., 2013), especially in neonatal animals – which have higher amounts of gap junctions present between various cell types than older animals (Ikeda et al., 2007). Intact bladder strips represent a more complex system where studying the muscle contractility is under the influence of an array of effects from the mucosa layer, including the mucosal interstitial cells, and urothelium, and the local release of neurotransmitters like ATP (Killi et al., 2014; Sui et al., 2014). Experiments in the isolated whole organ allow a physiological evaluation of phasic activity of the organ devoid of extrinsic innervation, which suppresses myogenic responses (Sadananda et al., 2013). They also allow study of bladder wall movements during contractions (Chun et al., 1989). However, despite providing an array of information, *in vitro* strips and whole organ recordings do not give a complete picture of whole organ behaviour in a context of a voiding animal (Drake et al., 2003a; Kullmann et al., 2014), notably because experimental methods to monitor the entire bladder surface at any given time have not yet been reported.

The translation from simpler muscle-only models to the whole organ experimental approach gives a broader view on phasic activity present in the bladder. Simultaneous visual inspection of video recordings and the pressure trace demonstrated that each pressure fluctuation is a composite net effect of all the elements making up the bladder wall motility. Overall bladder wall motility is a sum of motile and non-motile areas generating movement and/or maintaining the bladder wall tone. The effects of 18β-GA in isolated whole organ were independently identified using video visual inspection. The bladder overall must possess enough tone for these movements to manifest as detectable change in pressure. Changes in overall distance between points observed reflect shifting of the wall tension around the bladder upon exposure to 18β-GA and isoprenaline.

The intravesical pressure reflects the net sum of all wall movements, including local shortenings and local elongations, which have been recorded previously in adult mouse, rat, guinea pig and pig bladders (Coolsaet et al., 1993; Drake et al., 2003a,b; Lagou et al., 2006b). Although some pressure changes correspond to the localised contractions recorded, other wall microcontractions are also influencing the pressure recordings, further supporting the notion that a pressure change is a summation of all microcontractions and elongations on the bladder wall (Coolsaet et al., 1993; Vahabi and Drake, 2015). Decrease in baseline pressure as observed in isolated whole organ indicates relaxation of the muscle and can manifest in a change in bladder tone and a decrease in movement propagation, even though movement initiation is not eliminated by isoprenaline.

Although contractile events must be responsible for pressure change, some observed fluctuations in pressure did not have an associated micromotion. We surmise that further focuses of initiation causing pressure transients were present contralaterally, acting independently of those on the observed side of the bladder. This can be deduced from previous observations in the pig bladder, where areas contracting in phase and in anti-phase were often separated by considerable distances (Coolsaet et al., 1993). Longitudinal movement was observed to propagate, but this not evident in the transverse orientation, which may well reflect the anatomical arrangement of muscle (Hashitani et al., 2001; Hashitani and Brading, 2003). This suggests that using longitudinal strips in the rat better reflects contractile properties of the bladder than using transverse strips.

It must be noted that it is probably an oversimplification to regard tone, micromotion propagation and micro-contraction initiation as separate parameters. They are interlinked and at this stage there is no pharmaceutical agent which purely affects only one parameter independent of the others. In addition, the specific sites of action of agents used for pharmacological characterisation in this study are uncertain. Gap junctions and adrenoceptors are expressed on a range of bladder cells. In this study, micromotions in only a portion of the bladder wall were examined. In order to gain a comprehensive understanding of the pressure generation, it would be ideal to monitor the entire bladder surface for micromotions and inactive areas and summate their affects. At this stage, the technology to deliver such a global assessment of bladder contractility is not yet available.

### Clinical Implications

Such observations may well have clinical relevance; it may be possible to dissociate micromotion initiation, micro-contraction propagation, and overall tone and identify circumstances that could reflect known clinical features. For example, DO presumably reflects a high degree of initiation and propagation and high overall tone. Overactive bladder in the absence of DO could reflect a high degree of micromotion initiation, but low levels of propagation or low overall tone; in this case, the micromotions could cause distortions of the bladder wall responsible for stimulating sensory nerves but have modest effect on the detrusor pressure due to the dissipation of force. Since acetylcholine may be a factor initiating micromotions, this may explain why many people respond to antimuscarinic therapy. Likewise, the β3-adrenoceptor agonist mirabegron may be beneficial by a rather different effect, the reduction of overall tone. In theory, an ideal treatment would block spontaneous generations of micromotions during the storage phase, but without blocking triggered micromotions elicited by the efferent innervation responsible for detrusor contraction in voiding. Urodynamic assessment, by measuring only the summated pressure response, is unable to give a clear picture of bladder contraction activity and has to be interpreted accordingly.

## Conclusion

The findings shed light on the relationship between bladder micromotions and vesical pressure fluctuations. We observed congruent alterations in the amplitude of movements and pressure fluctuations when we modulated propagation of micromotions with gap junction blockade, and tone with adrenoceptor agonism. It was possible to see movement sometimes with no associated pressure fluctuations. Initiations of spontaneous micromotions is not gap junction dependent and persists during bladder relaxation but is inhibited by blocking Cl_Ca_ channels. Micro-contractions became less frequent, propagated over shorter distances, and tone was reduced by isoprenaline, with an evident reduction in the amplitude of pressure recordings. Thus, these recordings identify that the initiation and propagation of micromotions, and overall bladder tone lead to a summation of micro-contraction and force dissipation which underpins the consequent effect on intravesical pressure. At any given moment, the intravesical pressure is an integration of the bladder wall behaviour, comprising the initiation of micromotions, the extent of their propagation and the overall bladder tone.

## Author Contributions

BV, FC, AP, and MD contributed to the conception and design of the study. BC, DB, and MD acquired, analysed or interpreted the data. BC and DB generated the figures. BC, BV, FC, AK, AP, CF, and MD wrote or contributed to the writing of the manuscript. All authors contributed to manuscript revision, read and approved the submitted version.

## Conflict of Interest Statement

The authors declare that the research was conducted in the absence of any commercial or financial relationships that could be construed as a potential conflict of interest.

## Abbreviations

18β: glycyrrhetinic acid
ATP: adenosine triphosphate
CCh: calcium activated chloride channel (Cl_Ca_ channel) carbachol
CBX: carbenoxolone
DO: detrusor overactivity
DMSO: dimethyl sulfoxide, 18β-GA
SEM: standard error of the mean
