# Supplementary material for: Modulation of Bladder Wall Micromotions Alters Intravesical Pressure Activity in the Isolated Bladder
Source: Front Physiol. 2019 Jan 10;9:1937. doi: 10.3389/fphys.2018.01937 (PMC6335571; doi:10.3389/fphys.2018.01937)
Supplement: Supplementary file 1 [file Data_Sheet_1.PDF]

Supplementary Material

Modulation of Bladder Wall Micromotions Alters Intravesical Pressure Activity in the Isolated Bladder

Basu Chakrabarty[1†], Dominika A. Bijos[2,3†], Bahareh Vahabi[4], Francesco Clavica[5], Anthony J. Kanai[6], Anthony E. Pickering[1,2], Christopher H. Fry[1], Marcus J. Drake[2,3*]

*** Correspondence:** Marcus J. Drake: marcus.drake@bristol.ac.uk

1 Supplementary Data

Rhythmic contractions induced with CCh in denuded and intact strips had a greater amplitude and a more consistent frequency compared to control spontaneous contractions (Student's unpaired t-test, $p<0.05$) (Figure S1).

2 Supplementary Figures and Tables

2.1 Supplementary Figures

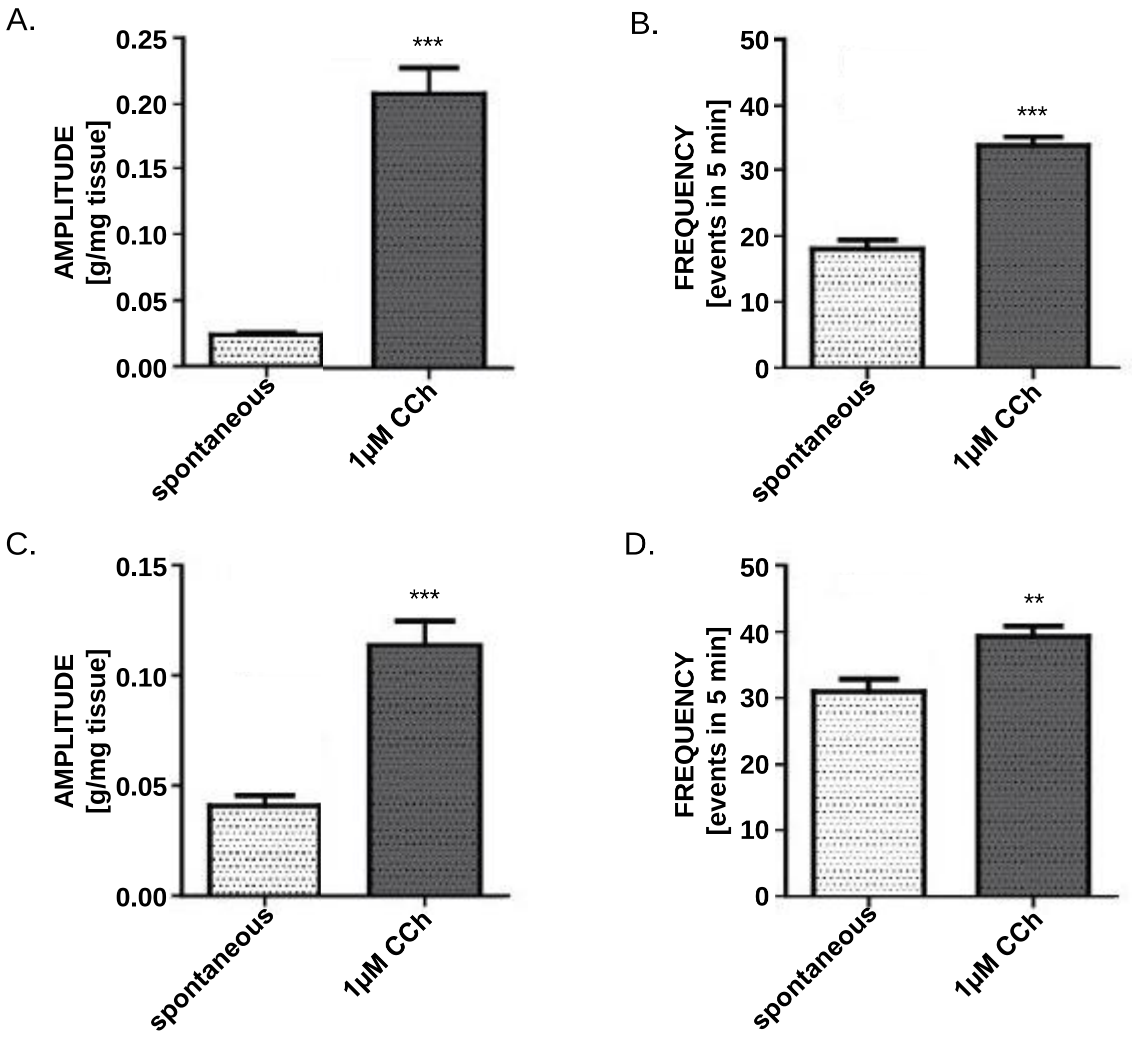

Supplementary Figure 1. The **(A)** amplitude and **(B)** frequency of spontaneous and CCh-induced contractions in denuded strips. The **(C)** amplitude and **(D)** frequency of spontaneous and CCh-induced contractions in intact bladder strips. ** $P < 0.01$, *** $P < 0.001$; Student's unpaired t-test.
